# A Novel Network-Based Computational Model for Prediction of Potential LncRNA–Disease Association

**DOI:** 10.3390/ijms20071549

**Published:** 2019-03-28

**Authors:** Yang Liu, Xiang Feng, Haochen Zhao, Zhanwei Xuan, Lei Wang

**Affiliations:** 1 College of Computer Engineering & Applied Mathematics, Changsha University, Changsha 410000, China; y1006480772@163.com (Y.L.); fengxiang@xtu.edu.cn (X.F.); 2 Key Laboratory of Hunan Province for Internet of Things and Information Security, Xiangtan University, Xiangtan 411100, China; zhaohc940702@163.com (H.Z.); zhanwei_xuan@163.com (Z.X.)

**Keywords:** lncRNA, disease, association prediction, resource allocation, label propagation

## Abstract

Accumulating studies have shown that long non-coding RNAs (lncRNAs) are involved in many biological processes and play important roles in a variety of complex human diseases. Developing effective computational models to identify potential relationships between lncRNAs and diseases can not only help us understand disease mechanisms at the lncRNA molecular level, but also promote the diagnosis, treatment, prognosis, and prevention of human diseases. For this paper, a network-based model called NBLDA was proposed to discover potential lncRNA–disease associations, in which two novel lncRNA–disease weighted networks were constructed. They were first based on known lncRNA–disease associations and topological similarity of the lncRNA–disease association network, and then an lncRNA–lncRNA weighted matrix and a disease–disease weighted matrix were obtained based on a resource allocation strategy of unequal allocation and unbiased consistence. Finally, a label propagation algorithm was applied to predict associated lncRNAs for the investigated diseases. Moreover, in order to estimate the prediction performance of NBLDA, the framework of leave-one-out cross validation (LOOCV) was implemented on NBLDA, and simulation results showed that NBLDA can achieve reliable areas under the ROC curve (AUCs) of 0.8846, 0.8273, and 0.8075 in three known lncRNA–disease association datasets downloaded from the lncRNADisease database, respectively. Furthermore, in case studies of lung cancer, leukemia, and colorectal cancer, simulation results demonstrated that NBLDA can be a powerful tool for identifying potential lncRNA–disease associations as well.

## 1. Introduction

In recent years, accumulating evidence studies have shown that non-coding RNAs (ncRNAs) are involved in various biological processes in the human body [1,2,3], and particularly long non-coding RNAs (lncRNAs), as a class of important heterologous ncRNAs with a length greater than 200 nt, play critical roles in various human biological processes such as chromatin modification, cell differentiation, proliferation and apoptosis, translational and post-translational regulation, and so on [4,5,6]. Moreover, mutation and disorder of lncRNAs may cause a broad range of complex human diseases [6,7]. For example, researchers have found that lncRNA-UCA1 will be expressed at high levels in lung cancer, bladder cancer, breast cancer, and colorectal cancer [8]. LncRNA HOTAIR can promote the malignant growth of human liver cancer stem cells by downregulating SETD2 in liver cancer stem cells [9]. Hence, detecting potential lncRNA–disease associations can not only help us understand the pathogenesis of human diseases at the molecular level, but also further facilitate the diagnosis, treatment, and prevention of human diseases [10].

Currently, with the rapid development of bioinformatics, some lncRNA–disease association databases such as LncRNADisease [11] and Lnc2Cancer [12] have been established successively. However, the number of known lncRNA–disease associations in these databases is far from meeting the needs of modern medical researches, due to traditional biological experiment methods for discovering potential relationships between lncRNAs and diseases that are very expensive and time-consuming [13]. Therefore, more and more researchers have devoted efforts to constructing computational models to identify potential relationships between lncRNAs and diseases. For instance, Chen and Yan [14] proposed a semi-supervised learning method called LRLSLDA to identify possible associations between lncRNAs and diseases. Yu et al. [15] presented a computational model which they called NBCLDA based on the naive Bayesian classifier to explore potential relationships between lncRNAs and diseases. In contrast to the above machine learning-based models, according to the assumption that functionally similar lncRNAs show similar interaction patterns with similar diseases, Sun et al. [16] proposed a computational model, RWRlncD, in which a global network was constructed first based on disease similarity, lncRNA functional similarity, and known lncRNA–disease associations, and then a random walk with restart method was implemented on the newly constructed global network to infer potential lncRNA–disease associations. Yao et al. [17] proposed a new computational model called LncPriCNet, in which a heterogeneous random walk was designed on a multi-layer composite network consisting of genes, lncRNAs, phenotypes, and associations between them to prioritize lncRNAs that are potentially associated with diseases. In all the above random walk-based models, it is obvious that only known lncRNA–disease associations are considered. In contrast to that, based on known lncRNA–miRNA and miRNA–disease associations, Chen [18] proposed a novel computational model called HGLDA to calculate potential association probabilities between lncRNAs and diseases, in which a hypergeometric distribution test was applied for each lncRNA–disease pair to indicate whether the lncRNA and disease significantly shared common miRNAs. Zhao et al. [19] developed a distance correlation set-based computational model, DCSMDA, to predict potential miRNA–disease associations, in which a tripartite miRNA–lncRNA–disease network was constructed through integrating disease similarity, miRNA similarity, and lncRNA similarity.

Inspired by the above-mentioned state-of-the-art methods, a network-based computational model NBLDA was proposed for this paper to predict potential lncRNA–disease associations based on the assumption that functionally similar lncRNAs show similar interaction patterns with similar diseases. In NBLDA, two new networks were constructed first based on known lncRNA–disease associations and Gaussian interaction profile kernel similarity for lncRNAs and diseases, and then we assigned an attraction that is proportional to *k^β^* to each node in the network, where *k* is the degree of the node and *β* is a freely adjustable parameter. Moreover, considering that traditional mass diffusion-based algorithms focused on unidirectional mass diffusion only, we further applied a consistence-based mass diffusion algorithm via bidirectional diffusion on NBLDA to predict potential lncRNA–disease associations by adopting a label propagation algorithm. Finally, in order to estimate the prediction performance of NBLDA, the framework of leave-one-out cross validation (LOOCV) was implemented, and simulation results show that NBLDA can achieve reliable AUCs of 0.8846, 0.8273, and 0.8075 in LOOCV based on three versions of known lncRNA–disease association datasets downloaded from the lncRNADisease database, respectively, which demonstrates the excellent prediction performance of NBLDA. In addition, in case studies of lung cancer, leukemia, and colorectal cancer, simulation results show that there are 9, 10, and 7 out of the top 10 predicted disease-related lncRNAs of these three kinds of diseases having been validated by evidence from studies in the PubMed literature and Lnc2Cancer database, respectively, which further indicates NBLDA has a satisfactory prediction performance in discovering potential lncRNA–disease associations as well.

## 2. Results

### 2.1. Performance Evaluation

In order to estimate the prediction performance of NBLDA, and described in this section, we implemented LOOCV on NBLDA based on known lncRNA–disease associations downloaded from the LncRNADisease database. While implementing LOOCV, each known lncRNA–disease association was left out in turn as a test sample and the other remaining known lncRNA–disease associations were taken as training samples. Moreover, all lncRNA–disease pairs without known relevance evidences were considered as candidate samples. Thereafter, we obtained the ranking of each test sample within all candidate samples according to their scores predicted by NBLDA, and then, the test sample was regarded as successfully predicted if its ranking exceeded a given threshold. Furthermore, the receiver operating characteristic (ROC) curves were drawn based on true positive rate (TPR, sensitivity) and false positive rate (FPR, 1-specificity) obtained at different thresholds. Here, the sensitivity represents the proportion of test samples with a ranking higher than the given threshold to all positive samples, whereas 1-specifcity indicates the ratio between candidate samples with a ranking above a given threshold and all candidate samples. Then, the areas under the ROC curve (AUCs) were further calculated to evaluate the predictive performance of our model NBLDA, and it is obvious that the larger the value of AUC, the better the prediction performance of NBLDA will be.

**Figure 1:**
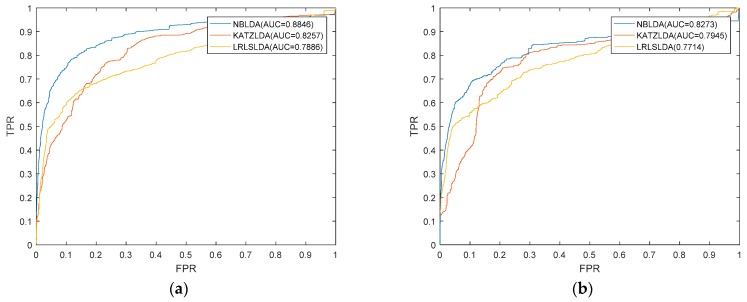
We compared the prediction performance of NBLDA with two classical methods for lncRNA-disease association prediction (KATZLDA and LRLSLDA). (**a**) Areas under the ROC curve (AUCs) achieved by NBLDA, KATZLDA, and LRLSLDA based on the dataset of *DS*_1_; (**b**) AUCs achieved by NBLDA, KATZLDA, and LRLSLDA based on the dataset of *DS*_2_.

**Figure 2:**
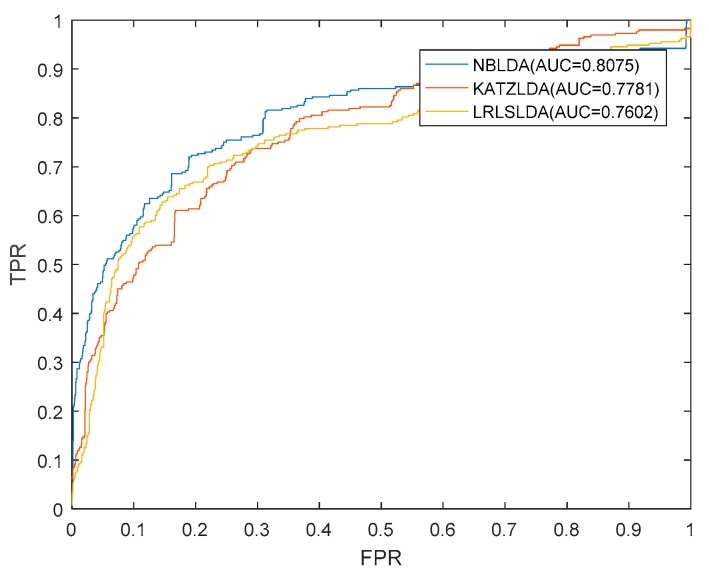
AUCs achieved by NBLDA, KATZLDA, and LRLSLDA based on the dataset of *DS*_3_.

We implemented NBLDA on three kinds of datasets under the framework of LOOCV. Moreover, we compared NBLDA with two state-of-the-art computational models such as KATZLDA [20] and LRLSLDA [14] on these three same datasets. Here, KATZLDA is a KATZ measurement model for lncRNA–disease association prediction based on known lncRNA–disease associations, disease similarity, and lncRNA similarity. LRLSLDA is a semi-supervised model that used Laplacian regularized least squares to predict potential lncRNA–disease associations by incorporating lncRNA expression profiles. As a result, NBLDA, KATZLDA, and LRLSLDA achieved AUCs of 0.8846, 0.8257, and 0.7886 on *DS*_1_, respectively (Figure 1a), AUCs of 0.8273, 0.7945, and 0.7714 were obtained on *DS*_2_, respectively (Figure 1b), and AUCs of 0.8075, 0.7781, and 0.7602 were obtained on *DS*_3_, respectively (Figure 2). It is obvious that our model NBLDA had better prediction performance than KATZLDA and LRLSLDA in LOOCV on both of these three kinds of datasets. In addition, during simulation, we found that the best AUCs were obtained at β=−0.1, which indicates that reducing the attractions of nodes with higher degrees can further improve the prediction accuracy of our model NBLDA, and this conclusion is consistent with previous studies [21].

### 2.2. Case Studies

**Figure 3:**
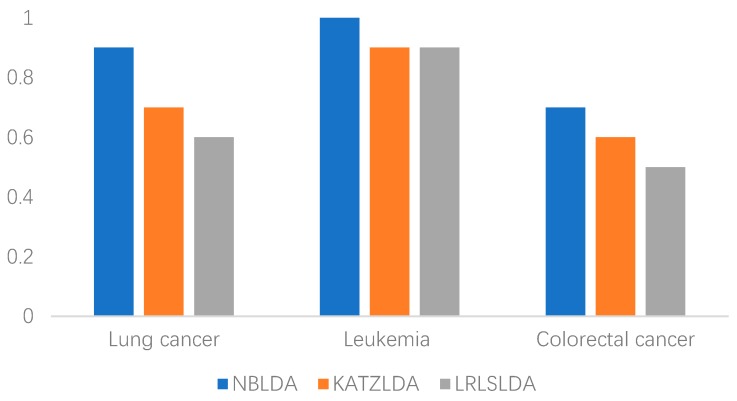
The accuracy of the top 10 related lncRNAs for lung cancer, leukemia, and colorectal cancer predicted by NBLDA, KATZLDA, and LRLSLDA, respectively.

Currently, cancer is one of the leading causes of human death worldwide, and is also a problem that modern medicine has not yet overcome [22,23,24]. To further evaluate the predictive performance of NBLDA, we implemented the case studies of lung cancer, leukemia, and colorectal cancer described in this section. During simulation, for any given investigated disease, those related known lncRNA–disease associations in *DS*_1_ were used as training samples for model learning. As a result, we list in Table 1 the top 10 disease-related lncRNAs predicted by NBLDA and the evidence to support these predicted results provided by the Lnc2Cancer database and the studies in the PubMed literature. Moreover, we show the accuracy of the top 10 related lncRNAs for the three diseases predicted by NBLDA, KATZLDA, and LRLSLDA, respectively (Figure 3). It is worthwhile to emphasize that only the lncRNA–disease pairs not included in *DS*_1_ were considered as verification candidates for simulation in our case studies.

Lung cancer is one of the most common cancers in the world with extremely high morbidity and mortality rates [25]. Over the past 50 years, the morbidity rate and the mortality rate of lung cancer have significantly increased in many countries, and these rates for male patients are the first among all malignant tumors [26,27]. In particular, the five-year survival rate for lung cancer patients is only about 15%, and about 1.4 million people die of lung cancer each year [28]. In order to better and more effectively promote the treatment of lung cancer, more and more studies have focused on the deregulation of protein-coding genes to identify oncogenes and tumor suppressors [29]. Recent studies have shown that lncRNAs are important for the development and progression of lung cancer [30]. We implemented NBLDA to reveal possible lung cancer-associated lncRNAs and, as illustrated in Table 1, simulation results show that there are 9 out of the top 10 predicted lncRNAs having been validated by the Lnc2Cancer database and related studies in the literature. For example, lncRNA PVT1 was expressed at high levels in lung cancer cells, which promoted proliferation of non-small cell lung cancer cells by regulating LATS2 expression [31]. LncRNA NEAT1 expression was significantly upregulated in lung cancer cells, and NEAT1 significantly accelerated tumor growth in vivo [32]. LncRNA TUG1 was expressed at low levels in lung cancer cells, which is involved in lung cancer cell growth by regulating LIMK2b via EZH2 [33].

Leukemia is a malignant clonal disease of hematopoietic stem cells, characterized by the ability of embryonic cells to self-renew, continuously proliferate, and escape apoptosis which ultimately inhibits the normal hematopoietic function of the human body [34,35]. In recent years, the prognosis of leukemia patients has greatly improved. However, the five-year survival rate of patients is still very low due to the high recurrence rate [36], and a more effective treatment method is urgently needed for patients. In recent years, in-depth molecular identification has completely changed our understanding of the mutations that drive disease, and related studies have shown that lncRNA plays a key role in the occurrence and development of leukemia [11]. We applied NBLDA to predict possible leukemia-associated lncRNAs and, as a result, there are 10 out of the top 10 predicted lncRNAs having been successfully confirmed by the Lnc2Cancer database and related studies in the literature (see Table 1). For example, lncRNA H19 expression was significantly upregulated in bone marrow samples from leukemia patients, which regulated ID2 expression by competitive binding to hsa-miR-19a/b [37]. The expression level of lncRNA MALAT1 was upregulated in acute myeloid leukemia, and MALAT1 knockdown in lung cancer cells led to upregulation of miR-101-3p expression, and then miR-101-3p reduced myeloid cell leukemia 1 (MCL1) expression by binding to 3’-UTR. [38]. LncRNA HOTAIR was expressed at high levels in leukemia patients, which promoted an increase in the number of white blood cells and a decrease in the number of hemoglobin and platelets, and its overexpression indicated a poor prognosis in patients [39].

Colorectal cancer (CRC) is one of the most common types of cancer in the United States and the second leading cause of cancer death [40]. The average lifetime risk of developing the disease in the United States is as high as 6% and the percentage of young patients is increasing [41]. With the development of medical technology, the mortality rate of patients with CRC has decreased but it is not satisfactory enough. Recent studies have shown that lncRNAs can be used as potential biomarkers for improving treatment efficacy of CRC [42]. A case study of CRC was implemented on NBLDA to identity potential associated lncRNAs. As illustrated in Table 1 above, it is easy to see that there are 7 out of the top 10 predicted lncRNAs having been validated to have associations with CRC based on the Lnc2Cancer database and the studies in the PubMed literature. For example, lncRNA CCAT2 was expressed at high levels in patients with colorectal cancer. At the same time, knockdown of CCAT2 could induce apoptosis and inhibit cell proliferation, which was a potential therapeutic target for CRC [30,43]. LncRNA XIST could promote the proliferation of CRC cells and act as an oncogene in CRC by targeting miR-132-3p, and its expression level was upregulated in both CRC tissue samples and CRC cells [44]. LncRNA BCYRN1 played an oncogenic role in CRC cells by upregulating NPR3 expression levels. Therefore, BCYRN1 could be used as a promising prognostic biomarker for CRC [45].

## 3. Discussion

Accumulating evidence studies have shown that lncRNAs are closely related to a variety of biological processes. Identifying potential lncRNA–disease association not only helps us understand the pathogenesis of disease at the molecular level of lncRNA, but also contributes to the diagnosis, treatment, prognosis, and prevention of diseases. In this paper, we presented a computational model NBLDA to reveal potential lncRNA–disease associations based on known lncRNA–disease associations and Gaussian interaction profile kernel similarity for lncRNAs and diseases. We improved the baseline algorithm of bipartite network recommendation based on the network topological similarity of the lncRNA–disease association network and resource allocation strategy of unequal allocation and unbiased consistence. A label propagation algorithm was then used to predict potential lncRNA–disease associations. NBLDA achieved AUCs of 0.8846, 0.8273, and 0.8075 in the validation framework of LOOCV based on three versions of known lncRNA–disease association datasets, which significantly improved the previous classic models. Furthermore, we conducted case studies of lung cancer, leukemia, and colorectal cancer, and simulation results show that there are 9, 10, and 7 out of the top 10 predicted candidate lncRNAs having been confirmed by previous studies in the literature respectively. As a result, both cross validation and case studies have shown that NBLDA has a good performance in potential lncRNA–disease association prediction.

The novel and reliable performance of NBLDA is mainly attributed to the following aspects. First, the method proposed by us is based on a classical approach that has already achieved excellent performance in predicting associations in other biological networks. Second, considering that the lncRNAs (or diseases) which are not associated with a given disease *D* (or a given lncRNA *L*) may also contribute resources to *D* (or *L*), we then constructed novel networks based on known lncRNA–disease associations and the Gaussian interaction profile kernel similarity for diseases and lncRNAs. Third, we adopted a resource allocation strategy of unequal allocation and unbiased consistence. Certainly, there are still some limitations in NBLDA which must be improved in the future. First of all, the similarity measures for diseases and lncRNAs are relatively simple, and more effective similarity measures such as disease semantic similarity, disease phenotypic similarity, and lncRNA functional similarity can improve the performance of our model. Moreover, although the numbers of lncRNA–disease associations data have increased compared to before, the known lncRNA–disease associations in our dataset are still too sparse, and the performance of NBLDA can be further improved when more lncRNA–disease associations datasets are available and more reliable types of biological datasets are integrated. Last but not least, increasing lncRNA–disease association data can be used as training samples for model learning with the development of biological experimental techniques.

## 4. Materials and Methods 

### 4.1. Human lncRNA–Disease Associations

Three versions of the datasets were downloaded from the LncRNADisease database (http://www.cuilab.cn/lncrnadisease), respectively (see Supplementary materials). First, we downloaded the 2017 version of the dataset (denoted as *DS*_1_) from the LncRNADisease database, and after removing duplicated records and associations that do not belong to human beings, we finally obtained 1695 known lncRNA–disease associations involving 314 diseases and 828 lncRNAs. Next, we downloaded the 2015 version of the dataset (denoted as *DS*_2_) from the LncRNADisease database, and after removing duplicated data, we finally obtained 621 known lncRNA–disease associations including 226 diseases and 285 lncRNAs. Finally, we downloaded the 2012 version of the dataset (denoted as *DS*_3_) from the LncRNADisease database, and after removing duplicated data, we finally obtained 293 known lncRNA–disease associations including 167 diseases and 118 lncRNAs. Thereafter, we adopted an adjacency matrix *Y* to indicate known associations between lncRNAs and diseases. In the adjacency matrix *Y*, if there is a known association between lncRNA *l_i_* and disease *d_j_*, then there is *Y*(*i,j*) = 1; otherwise, there is *Y*(*i,j*) = 0. Moreover, for convenience, we further introduced *N_D_* and *N_L_* to denote the number of diseases and lncRNAs collected above, respectively.

### 4.2. Gaussian Interaction Profile Kernel Similarity for lncRNAs and Diseases

Based on the hypothesis that functionally similar lncRNAs are always associated with similar diseases [46], for any given lncRNAs *l_i_* and *l_j_*, we can obtain the Gaussian interaction profile kernel similarity between *l_i_* and *l_j_* according to the topologic information of known lncRNA–disease association network as follows:

(1) $$S_{l}\left(l_{i},l_{j}\right)=\exp\left(-\gamma_{l}{\left|\left|IP\left(l_{i}\right)-IP\left(l_{j}\right)\right|\right|}^{2}\right),$$

(2) $$\gamma_{l}=\gamma_{l}{}^{\prime }/\frac{1}{N_{L}}\sum_{i=1}^{N_{L}}{\left|\left|IP\left(l_{i}\right)\right|\right|}^{2},$$

where *IP*(*l_i_*) is the *i*th row of the adjacency matrix *Y* and represents the interaction profile of lncRNA *l_i_* with all diseases. The parameter $\gamma_{l}$ is used to control the Gaussian kernel bandwidth, and $\gamma_{l}{}^{\prime }$ is a bandwidth parameter that will be set to 1 according to previous work [47]. Obviously, according to Equation (1) above, we can obtain a similarity matrix *S_l_* based on these lncRNAs collected above.

In a similar way, for any given diseases *d_i_* and *d_j_*, we can obtain the Gaussian interaction profile kernel similarity between *d_i_* and *d_j_* according to Equation (3) as follows:

(3) $$S_{d}\left(d_{i},d_{j}\right)=\exp\left(-\gamma_{d}{\left|\left|IP\left(d_{i}\right)-IP\left(d_{j}\right)\right|\right|}^{2}\right),$$

(4) $$\gamma_{d}=\gamma_{d}{}^{\prime }/\frac{1}{N_{D}}\sum_{i=1}^{N_{D}}{\left|\left|IP\left(d_{i}\right)\right|\right|}^{2},$$

where *IP*(*d_i_*) is the *i*th column of the adjacency matrix *Y* and represents the interaction profile of disease *d_i_* with all lncRNAs. The parameter $\gamma_{d}$ is used to control the Gaussian kernel bandwidth and $\gamma_{d}{}^{\prime }$ is set to 1 [47]. Obviously, according to Equation (3) above, we can obtain a similarity matrix *S_d_* based on these diseases collected above.

### 4.3. Prediction Model of NBLDA

**Figure 4:**
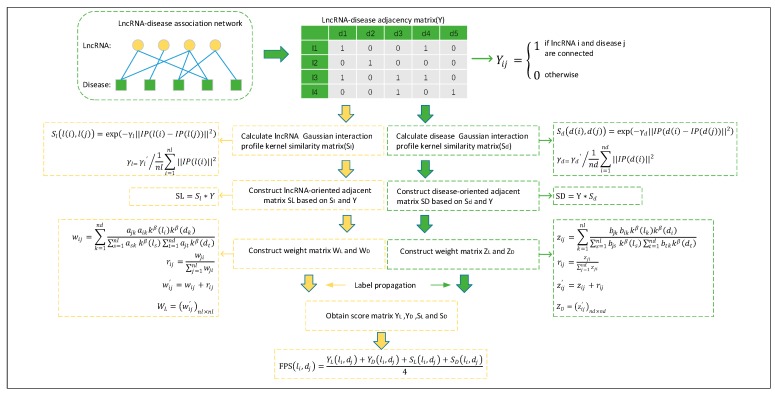
Flowchart of NBLDA, in which the weighted matrix *W*_D_ and *Z*_L_ can be calculated in a similar way as *Z*_D_ and *W*_L_, respectively.

As illustrated in Figure 4, we can model the prediction problem of potential lncRNA–disease association as the problem of resource allocation on the lncRNA–disease bipartite network. According to the assumption that functionally similar lncRNAs tend to show similar interaction patterns with similar diseases [46], it is reasonable to deduce that each lncRNA (or disease) should contribute resources to a specific disease (lncRNA) along with its similar lncRNAs (diseases). Therefore, we can construct a matrix $SL={\left\{a_{ij}\right\}}_{N_{L}\times N_{D}}$ and a matrix $SD={\left\{b_{ij}\right\}}_{N_{L}\times N_{D}}$ based on the matrices *S_l_*, *S_d_*, and *Y* as follows:

(5) $$SL=S_{l}*Y,$$

(6) $$SD=Y*S_{d}.$$

Obviously, according to the matrix *SL*, we can construct a bipartite network first, and then, for a randomly given node *ψ* in the newly constructed bipartite network, supposing that *ψ* has been assigned an attraction such as *k^β^*(*ψ*), where *k*(*ψ*) represents the degree of node *ψ* in the bipartite network and *β* is a freely adjustable parameter, it is obvious that *β* = 0 means the average allocation of resources, *β* < 0 means that nodes with lower degrees are more attractive and will obtain more resources, and *β* > 0 indicates that nodes with higher degrees have greater attraction and will be allocated more resources [21]. Thus, in general, the resource allocation based on the matrix *SL* can be divided into the following processes:

First, in the newly constructed bipartite network, each lncRNA node will allocate resources to its neighboring disease nodes based on the attractions of its neighboring disease nodes. Here, for a given lncRNA node, its neighboring disease nodes denote all disease nodes that have associations in *SL* with this given lncRNA node, that is, all these disease nodes that have direct edges with this given lncRNA node in the bipartite network. Thus, for a given lncRNA node *l_j_* and one of its neighboring disease node *d_k_*, the resource *p_jk_* that the disease node *d_k_* will obtain from the lncRNA node *l_j_* can be calculated as follows:

(7) $$p_{jk}=\frac{a_{jk}k^{\beta }\left(d_{k}\right)}{\sum_{t=1}^{N_{D}}a_{jt}k^{\beta }\left(d_{t}\right)}.$$

Second, in a similar way as for the disease node *d_k_*, let the lncRNA node *l_i_* be one of its neighboring lncRNA nodes. Here, for a given disease node, its neighboring lncRNA nodes denote all lncRNA nodes that have associations in *SL* with this given disease node, that is, all these lncRNA nodes that have direct edges with this given disease node in the bipartite network, then the resource *q_ik_* that the lncRNA node *l_i_* will obtain from the disease node *d_k_* can be calculated as follows:

(8) $$q_{ik}=\frac{a_{ik}k^{\beta }\left(l_{i}\right)}{\sum_{s=1}^{N_{L}}a_{sk}k^{\beta }\left(l_{s}\right)}.$$

Finally, according to Equations (7) and (8) above, for any two given lncRNA nodes *l_i_* and *l_j_*, we can define the resources that *l_i_* will obtain from *l_j_* as follows:

(9) $$w_{ij}=\sum_{k=1}^{N_{D}}q_{ik}p_{jk}=\sum_{k=1}^{N_{D}}\frac{a_{jk}a_{ik}k^{\beta }\left(l_{i}\right)k^{\beta }\left(d_{k}\right)}{\sum_{s=1}^{N_{L}}a_{sk}k^{\beta }\left(l_{s}\right)\sum_{t=1}^{N_{D}}a_{jt}k^{\beta }\left(d_{t}\right)},$$

where *w_ij_* indicates the resource diffusion capability from *l_j_* to *l_i_*, that is, the probability that *l_i_* will be recommended because *l_j_* is selected by given disease. In addition, considering the consistency of capability that resources move in both directions [48], we further define the resource diffusion capability from *l_i_* to *l_j_* as follows:

(10) $$r_{ij}=\frac{w_{ji}}{\sum_{j=1}^{N_{L}}w_{ji}}.$$

Then, according to Equations (9) and (10) above, we can define the sum of contribution from resource allocation between *l_i_* and *l_j_* as follows:

(11) $$w_{ij}^{\prime }=w_{ij}+r_{ij}.$$

Hence, according to Equation (11) above, we can obtain a weighted matrix $W_{L}={\left(w_{ij}^{\prime }\right)}_{N_{L}\times N_{L}}$. Then, we can adopt the label propagation algorithm to predict potential lncRNA–disease associations based on the adjacency matrix *Y* and the weight matrix *W_L_*. First for any given disease node *d_i_* in the bipartite network, let *Y_i_* be the *i*th column of the adjacency matrix *Y*, then for convenience, we define the lncRNAs in *Y_i_* as the initial label information of *d_i_*. Next, in each iterative process, supposing that each lncRNA node will receive information from its neighboring nodes with probability *α* and keep its initial label information with probability 1 − *α*, we can then express the iterative process as follows:

(12) $$Y_{i}^{t+1}=\alpha W_{L}Y_{i}^{t}+\left(1-\alpha \right)Y_{i}^{0},$$

where $Y_{i}^{0}$ = *Y_i_* represents the interaction profile of disease *d_i_* with all lncRNAs before the beginning of the iterative process, and $Y_{i}^{t}$ represents the predicted label information of *d_i_* at the *t*th iteration. In addition, let $Y^{0}$ = *Y*, we can then further represent the iteration process in matrix form as follows:

(13) $$Y^{t+1}=\alpha W_{L}Y^{t}+\left(1-\alpha \right)Y^{0}.$$

According to Equation (13) above, we will keep updating the label matrix $Y^{t+1}$ until it converges to $Y_{L}$:

(14) $$Y_{L}=\left(1-\alpha \right){\left(I-\alpha W\right)}^{-1}Y^{0},$$

where $I\in R^{N_{L}\times N_{L}}$ is an identity matrix.

From the above descriptions, it is easy to see that *Y_L_* is an lncRNA-oriented lncRNA–disease association score matrix obtained based on the bipartite network that is constructed according to the matrix *SL*. In a similar way, it is obvious that we can obtain another disease-oriented lncRNA–disease association score matrix *Y_D_* based on the bipartite network constructed according to the matrix *SL*. Moreover, in a similar way, we can further obtain an lncRNA-oriented lncRNA–disease association score matrix *Z_L_* and a disease-oriented lncRNA–disease association score matrix *Z_D_* based on the bipartite network constructed according to the matrix *SD* as well. Subsequently, based on the above newly obtained matrices such as *Y_L_*, *Y_D_*, *Z_L_*, and *Z_D_*, and for convenience, let FPS(i,j), $Y_{L}\left(i,j\right)$, $Y_{D}\left(i,j\right)$, $Z_{L}\left(i,j\right)$, and $Z_{D}\left(i,j\right)$ denote $FPS\left(l_{i},d_{j}\right)$, $Y_{L}\left(i,j\right)$, $Y_{D}\left(l_{i},d_{j}\right)$, $S_{L}\left(l_{i},d_{j}\right)$, and $S_{D}\left(l_{i},d_{j}\right)$, respectively. We can then construct a final lncRNA–disease association score matrix *FPS* as follows:

(15) $$FPS\left(i,j\right)=\frac{Y_{L}\left(i,j\right)+Y_{D}\left(i,j\right)+S_{L}\left(i,j\right)+S_{D}\left(i,j\right)}{4},$$

where *i* ∈ [1, *N_L_*] and *j* ∈ [1, *N_D_*].

## Figures and Tables

**Table 1:** Top 10 potential lung cancer, leukemia, and colorectal cancer-related lncRNAs predicted by NBLDA and confirmations for these predicted associations provided by the Lnc2Cancer database and the studies in the PubMed literature.

Disease	LncRNA	Evidence (PMID)	Rank
Lung cancer	PVT1	26493997,28731781,28972861,27904703,29133127	1
Lung cancer	NEAT1	25818739,29152741,28295289,28615056,29095526	2
Lung cancer	TUG1	28069000,24853421,29277771,28121347,27485439	3
Lung cancer	XIST	29130102,29339211,26339353,29337100,28248928	4
Lung cancer	HULC	30575912	5
Lung cancer	LINC-ROR	28459375,28516515,29028092	6
Lung cancer	PANDAR	28121347,25719249	7
Lung cancer	MIAT	29487526,28843520,29228680,29795987,27981551	8
Lung cancer	HNF1A-AS1	27981551,29289833	9
Leukemia	H19	15645136,29703210,24685695,28765931,29643943	1
Leukemia	MALAT1	28713913	2
Leukemia	HOTAIR	27748863,26622861,27875938,25979172,26261618	3
Leukemia	MEG3	28407691,28190319,19595458,14602737,29029424	4
Leukemia	PVT1	29510227,26545364	5
Leukemia	GAS5	27951730	6
Leukemia	UCA1	27854515,29762824,26053097,29663500	7
Leukemia	TUG1	29654398	8
Leukemia	XIST	7981627	9
Leukemia	SNHG5	28861326,29917184	10
Colorectal cancer	CCAT2	29181105,27875818,28838211,26853146,23796952	1
Colorectal cancer	XIST	29495975,29137332,17143621,28730777,29484395	2
Colorectal cancer	BCYRN1	30114690	3
Colorectal cancer	HNF1A-AS1	28791380,29145164	4
Colorectal cancer	MIAT	29686537	5
Colorectal cancer	ATB	25750289	6
Colorectal cancer	TUSC7	27683121,28214867,23680400,28979678	10

## Supplementary Materials

Supplementary materials can be found at https://www.mdpi.com/1422-0067/20/7/1549/s1.

## Abbreviations

LOOCV	leave-one-out cross validation
FPS	final lncRNA–disease association score matrix
ROC	receiver operating characteristic
TPR	true positive rate
FPR	false positive rate
AUC	area under the ROC curve
